# *In vitro* disinfection, pH and solubility of bioceramic intracanal medication with calcium hydroxide

**DOI:** 10.1590/1807-3107bor-2025.vol39.124

**Published:** 2025-11-17

**Authors:** Larissa Braz PONTES, Camila Soares LOPES, Gisselle Moraima CHÁVEZ-ANDRADE, Jéssica Arielli PRADELLI, Guilherme Ferreira da SILVA, Mario TANOMARU-FILHO, Juliane Maria GUERREIRO-TANOMARU

**Affiliations:** (a) Universidade Estadual Paulista – Unesp, Araraquara School of Dentistry, Department of Restorative Dentistry, Araraquara, SP, Brazil.; (b) Centro Universitário Sagrado Coração, – Unisagrado, Department of Dentistry, Bauru, SP, Brazil.

**Keywords:** Calcium Hydroxide, Calcarea Silicata, Enterococcus faecalis

## Abstract

To evaluate the pH, solubility and intratubular disinfection of the bioceramic intracanal drug Bio-C^®^ Temp (BCT), calcium hydroxide-based paste Calen^®^ (CAL) and their associations in different proportions: BCT 75% + CAL 25%; BCT 50% + CAL 50%; BCT 25% + CAL 75%. Polyethylene tubes containing the medication, were used. The pH was evaluated after 1, 3, 7, 14 and 21 days and solubility after 14 days. Bovine dentin tubes were contaminated with *Enterococcus faecalis* to assess intratubular disinfection by using confocal laser scanning microscopy and use of Live/Dead BacLight Bacterial stain. Data were submitted to statistical tests of normality, then ANOVA and Tukey (α = 0.05). BCT showed a lower pH after 3 and 14 days (p < 0.05). CAL had the highest pH at all time intervals (p > 0.05). CAL and associations with BCT showed greater weight loss (p < 0.05). BCT showed intratubular disinfection similar to that of BCT/CAL 25% (p > 0.05). CAL and BCT/CAL at 50% and 75% were similar and had the highest intratubular disinfection values (p > 0.05). Combinations of Bio-C® Temp with 50% or more calcium hydroxide paste provide higher alkalinization, solubility and intratubular disinfection values of the bioceramic medication, favoring its potential for clinical application.

## Introduction

Endodontic treatment must control microorganisms in root canal systems and anatomical complexities.^1^ Intracanal medication is used to complement disinfection of the root canal preparation, thereby favoring the treatment prognosis.^2^

The Calcium silicate-based bioceramic sealer was developed as a root canal repair and filling material with adequate physicochemical, biological^3^, and bioactivity properties.^3,4^ Bio-C^®^ Temp (BCT; Angelus, Londrina, Brazil) was developed as a bioceramic intracanal medication for use in conventional endodontic treatment, retreatment, apexification and endodontic regeneration. According to the manufacturer it is an alternative to calcium hydroxide.^5^ BCT is a ready-to-use paste, based on calcium silicates, calcium aluminate, calcium oxide, radiopacifying agent calcium tungstate and titanium oxide, in addition to a resin based vehicle.^5^

BCT is biocompatible,^6^ radiopaque,^4,7^ has an alkaline pH,^4^ releases calcium ions (Ca^++^)^4,8^and has been shown to induce the deposition of mineralized nodules in alkaline phosphatase activity and mineralization (alizarin red) tests, thus demonstrating its bioactive potential.^9^ Furthermore, the residue of BCT in the root canal enhanced the bond strength to the bioceramic sealer, by creating an adhesive layer and preventing gaps.^10^ However, when compared with calcium hydroxide-based medication, BCT induced a coronal discoloration in the long term,^11^ showed a lower alkalinization capacity of the medium^4^ and a lower potential for antibacterial and anti-biofilm activity against *Enterococcus faecalis*.^9^

Calcium hydroxide (Ca(OH)_2_) is widely used as an intracanal medication due to its antibacterial activity and ability to induce the formation of mineralized tissue.^12^Ca(OH)_2_ has an alkaline pH because of dissociation and diffusion of hydroxyl ions (OH^-^) favoring antimicrobial efficacy.^13^ Calen® (CAL; SS White Dental Articles, Rio de Janeiro, Brazil) is an intracanal medication based on Ca(OH)_2_ associated with a viscous polyethylene glycol 400 vehicle.^14^ CAL is biocompatible,^6,15^releases Ca^++^ and OH^-^ ions, has an alkaline pH,^16^ antimicrobial activity,^13,17^ inactivates bacterial endotoxins^18^and induces mineralization.^19^

The association of a Ca(OH)_2_ (CAL) based paste with bioceramic intracanal medication (BCT) can provide a higher alkalinization potential and increase antimicrobial activity, without altering its bioactive potential, since this material has calcium silicate calcium, and its main feature is bioactivity.^3^ The study aim was to improve the bioceramic intracanal medication relative to its antimicrobial potential to obtain a medication with a higher number of favorable properties, thereby promoting adequate root canal system disinfection and stimulating periapical repair, to enable subsequent filling and evaluation of the repair in the long term. Moreover, this study aim was to evaluate the pH, solubility and intratubular disinfection of calcium silicate-based medication and their association with Ca(OH)_2_-based medication in different proportions. The null hypothesis was that the association of CAL with BCT would not modify its physicochemical properties and would achieve the intratubular disinfection of dentin contaminated with *E. faecalis*.

## Methods

The materials evaluated were calcium silicate-based medications (BCT), based on Ca(OH)_2_ (CAL), and their associations in different proportions (mass) (Table 1). The sample size was determined based on a pilot study. The calculation by software G*Power (Heinrich-Heine-Universitat) with a power of 0.8 and alpha error of 0.05. All tests were performed by a single, trained operator who was blind to the composition of the experimental groups.

**Table 1:** Experimental groups and their respective compositions and proportions.

Groups	Composition	Proportions (mass %)
Bio-C^®^ Temp (BCT)	Calcium Silicate, Calcium Aluminate, Calcium Oxide, Resin Base, Calcium Tungstate, and Titanium Oxide	BCT (100%)
Calen^®^ (CAL)	Calcium Hydroxide, Zinc Oxide, Colophonium, and Polyethylene Glycol 400	CAL (100%)
	1+ 2	
BCT/CAL 25%	1+ 2	BCT (75% + CAL 25%)
BCT/CAL 50%	1+ 2	BCT (50% + CAL 50%)
BCT/CAL 75%		BCT (25% + CAL 75%)

BCT: Bio-C® Temp; CAL: Calen®.

### pH

Polyethylene Tubes measuring 10 mm long and 1.6 mm in internal diameter were filled according to the experimental groups (n = 10). The tubes were immersed in 10 mL of distilled water and kept in an oven at 37^0^C. After time intervals of 1, 3, 7, 14, and 21 days the pH of the solution was determined at an ambient temperature of 25^0^C using a previously calibrated digital pH meter (FiveEasy Plus FP20, Mettler Toledo ®, Columbus, USA), The control group was based on the pH values of distilled water in which no samples were immersed.

### Solubility

Polyethylene tubes (n = 6) measuring 10 mm in length and 2 mm in internal diameter were filled with the medications and kept in an incubator at 37°C and 95% humidity for 24 hours. After this, samples were weighed on an electronic analytical balance (OHAUS Adventurer®, New Jersey, USA) to obtain the initial mass. Subsequently, the tubes were placed in plastic containers containing 7.5 mL of distilled water at 37°C for 14 days. After this period, the tubes were removed from the distilled water, placed in a desiccator and weighed on an electronic analytical balance every 24 hours until the final mass stabilized (approximately 14 days). The solubility of these medications was determined by the difference between the initial and final mass. This difference was converted into a percentage based on the initial weight (% mass loss).

### Intratubular disinfection

#### Preparation of dentin tubes

University Ethics Committee (no 35/2020) approved this study. Based on the methodology of Haapasalo et al.,^20^ single-root bovine teeth were sectioned at 1 and 4 mm from the cementoenamel junction to obtain dentin tubes measuring 3 mm long. The tubes were prepared with a Gates-Glidden drill number 4 (Dentsply Maillefer, Ballaigues, Switzerland) and Maxicut (American Burr, Pahoa, Brazil) to standardize the internal diameter at 1.1 mm and wall thickness at 2 mm. The specimens were submitted to an ultrasonic bath with 17% ethylenediaminetetraacetic acid (EDTA) to remove debris, sterilized in an autoclave at 121ºC for 20 minutes, and randomly divided according to the experimental groups and Control Group (Polyethylene glycol 400).

#### Dentin contamination

The procedures were performed in a laminar flow chamber (VecoFlow Lida, Campinas, Brazil). Before contamination, the samples were inserted into a culture medium tryptic soy broth (TSB) (Difco, Detroit, USA) and submitted to an ultrasonic bath for 15 minutes, to rehydrate and make it possible for the bacterial suspension to penetrate into the dentinal tubules. The specimens were inserted into Eppendorf type and 500µL of bacterial inoculum of *E. faecalis* (ATCC 29212) was added. The concentration of the inoculum was adjusted in a spectrophotometer at an optical density equivalent to 1x10^8^ CFU mL^-1^. The tubes were centrifuged at speeds of 1400g, 2000g, 3600g, and 5000g (Centrifuge 5430; Eppendorf, Hamburg, Germany) for 5 minutes, repeated twice for each centrifugation process.^21^ After centrifugation procedures, the samples were incubated at 37ºC in a TSB culture medium in a microaerophilic environment. These procedures were repeated after 48 hours, for 5 days, and the culture medium TSB was renewed daily.

#### Filling the specimens

After the incubation period, the tubes were removed from the Eppendorf, and their external surfaces were decontaminated with 2.5% Sodium Hypochlorite Solution (Asfer, São Caetano do Sul, Brazil) and neutralized with 1% Sodium Thiosulphate (Sigma, Barueri, SP, Brazil). The canal of the tubes irrigated with physiological solution, filled with 17% EDTA for 3 minutes, and then irrigated with physiological solution. The tubes were dried with sterile gauze and filled with the medications. The Control Group was filled with Polyethylene Glycol 400 (Sigma, Barueri, Brazil). The samples were kept in an oven at 37ºC with 95% humidity for 3 days.

#### Evaluation by confocal laser scanning microscopy (CLSM)

Phosphate-buffered saline (PBS) (Sigma, Barueri, Brazil) and #40 file (Dentsply Maillefer, Ballaigues, Switzerland) were used to remove the medication. The tubes were cleaved in an Isomet 1000 precision cutter (Buehler Ltd, Lake Bluff, USA) to obtain two longitudinal halves of each specimen. One half of each specimen was immersed in 17% EDTA for 3 minutes followed by sterile saline to remove the smear layer resulting from the cut. Samples (n = 6) received 30µL of Live/Dead BacLight Bacterial Viability solution (Molecular Probes, Inc, Eugene, USA) for 15 minutes. The kit includes two dyes: SYTO9, a green, fluorescent dye capable of penetrating living bacteria, and propidium iodide, a red fluorescent dye penetrating dead bacteria. The samples were taken to the confocal laser scanning microscope (Leica TCS-SPE; Leica Microsystems GmbH, Mannheim, Germany) to evaluate the intratubular disinfection activity at 20X magnification. Simultaneous dual-channel images were captured to visualize green (live cells) and red (dead cells) fluorescence, with excitation/emission wavelengths of 480/500 nm for SYTO9 and 490/635 nm for propidium iodide. The images were analyzed using the ZEN Lite Blue Software (Carl Zeiss, Jena, Germany). To standardize the area evaluated, an area of approximately 0.70 mm x 0.70mm was established for all images. The presence of viable and non-viable cells in this patterned area was automatically determined by the software, which calculated the intensity of the mean values of the green (live bacteria) and red (dead bacteria) areas. The percentage (%) of dead cells was calculated by the following equation: [% dead cells = red intensity/(red intensity + green intensity) x100].

#### Statistical analysis

The GraphPad Prism 6.01 program (GraphPad Software, Inc., La Jolla, USA) was used to evaluate pH, solubility and intratubular decontamination data. The data were submitted to the normality test and then to ANOVA and Tukey’s posttest with a significance level of 5%.

## Results

### pH

Medications promoted an alkaline pH in all time intervals, which were higher than those in the Control Group (p < 0.05). At 3 and 14 days, BCT had the lowest pH (p < 0.05) and in the remaining time intervals showed a pH similar to that of BCT/CAL 25% (p > 0.05). BCT/CAL 50% promoted an alkaline pH similar to that of CAL at 3 and 7 days (p > 0.05). BCT/CAL 75% showed pH similar to that of CAL in all time intervals (p > 0.05) (Table 2).

**Table 2:** Mean and standard deviation of the pH and solubility (% mass loss) values observed in the different groups.

Tests	BCT	BCT/CAL25%	BCT/CAL50%	BCT/CAL75%	CAL	Control
pH 1 day	10.5 ± 0.46^d^	10.76 ± 0.32^cd^	10.96 ± 0.23^bc^	11.3 ± 0.15 ^ab^	11.53 ± 0.12^a^	7.637 ± 0.24^e^
pH 3 days	9.906 ± 0.67^c^	10.74 ± 0.34^b^	11 ± 0.51 ^ab^	11.23 ± 0.29 ^ab^	11.4 ± 0.10^a^	7,79 ± 0,13^d^
pH 7 days	9.583 ± 0.92^c^	10.25 ± 0.68^bc^	10.49 ± 0.30 ^ab^	10.85 ± 0.38 ^ab^	11.12 ± 0.62^a^	7,468 ± 0,24^d^
pH 14 days	9,57 ± 0,37^d^	10.8 ± 0.38^c^	11.04 ± 0.59^bc^	11.4 ± 0.41 ^ab^	11.64 ± 0.19^a^	6.464 ± 0.28^e^
pH 21 days	10.41 ± 0.36^c^	10.66 ± 0.52^c^	10.83 ± 0.67^bc^	11.3 ± 0.19 ^ab^	11.58 ± 0.13^a^	6,464 ± 0,28^d^
Solubility	18.24 ± 0.69^b^	20.23 ± 1.47 ^ab^	21.72 ± 2.25^a^	22.92 ± 1.70^a^	21.84 ± 3.31^a^	------

BCT: Bio-C® Temp; CAL: Calen®. Different letters in the same line represent significant differences.

### Solubility

All the experimental groups exhibited a loss of mass. BCT showed the lowest loss of mass when compared with CAL and its associations with BCT in the different proportions (P < 0.05), except for BCT/CAL 25%. The highest loss of mass was observed in BCT/CAL 50%, BCT/CAL 75%, and CAL (P > 0.05) (Table 2).

### Intratubular disinfection

All the medications provided a higher percentage of dead cells (red areas) than the Control Group (p < 0.05). A higher proportion of dead cells (red areas) of *E. faecalis* was observed for CAL, BCT/CAL 50%, and BCT/CAL 75% (p < 0.05), which were similar among them (p > 0.05). There was no significant difference between BCT and BCT/CAL 25% (Table 3) (Figure).

**Table 3:** Mean and standard deviation of the percentage (%) values of non-viable cells of *E. faecalis* at 3 days after filling the tubes with the intracanal medications and control group.

BCT	BCT/CAL25%	BCT/CAL50%	BCT/CAL75%	CAL	Control
31.17±2.82^b^	34.46 ± 3.27^b^	38.66 ± 5.79 ^ab^	38.37 ± 5.01 ^ab^	40.69 ± 3.58^a^	14.12 ± 2.52^c^

BCT: Bio-C® Temp; CAL: Calen®; Different letters in the same line represent statistically significant differences.

**Figure:**
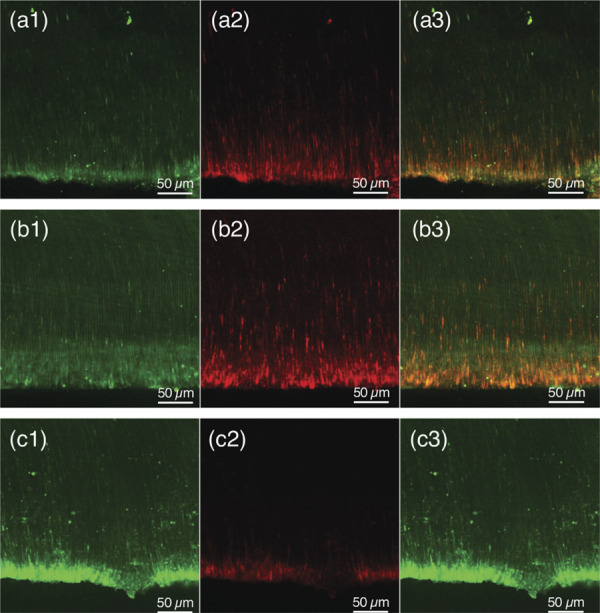
Images of confocal laser scanning microscopy of the dentin tubules contaminated with *E. faecalis* at 3 days after contact with the medications: (a) BCT/CAL 25%, (b) BCT/CAL 75% and (c) Control Group (Polyethylene glycol 400). 1) represents viable cells; 2) non-viable cells and 3) superimposition of the viable and non-viable cells.

## Discussion

The null hypothesis was partially rejected, as the association of BCT with 50% or more of the calcium hydroxide medication provided an increase in pH, solubility and intratubular decontamination against *E. faecalis* in comparison with the bioceramic medication.

Bioceramic materials generally show little antimicrobial activity when compared with calcium hydroxide-based materials, due to their composition and mechanism of action.^9^ The release of Ca^++^ and OH^-^ions by medication based on calcium silicates is relatively low compared with calcium hydroxide.^4,22^ These ions provide the medium with an alkaline pH and antimicrobial activity, but the concentration released is not sufficient to effectively eliminate an established endodontic infection.^9^However, it is important to emphasize that calcium silicate-based intracanal medication has other beneficial properties, such as biocompatibility^6^ and bioactivity, stimulating tissue repair.^9^ Therefore, bioceramic medication can play an important role in endodontic therapy when associated with other antimicrobial strategies, as proposed in this study.

The present study demonstrated that BCT has lower pH, solubility and antimicrobial activity when compared with CAL. A pH of between 8.6-10.3 is recommended for alkaline phosphatase activation and helps with the mineralization process.^23^ The pH of calcium hydroxide-based medications is approximately 12.5.^2^ BCT demonstrated lower pH than the Ca(OH)_2_-based mediation in Ultracal® XS aqueous vehicle.^4^ BCT bioceramic medication demonstrated a pH of approximately 8 when compared with calcium silicate materials.^4^The lower alkalinization potential of BCT may be related to the lower capacity to release Ca^++^ and OH^-^ ions than the Ca(OH)_2_-based medication.^4^As it may also be related to the different vehicles that determine properties such as dissociation and ionic release, which are essential factors in the biological behavior and antimicrobial activity of the materials.^4,24^However, further studies need to be conducted to confirm this.

The lower release of Ca^++^ and OH^-^ions from calcium silicate-based medication results from its chemical composition and physical-chemical properties. The calcium silicate contained in these medications has a stable crystalline structure, mainly composed of complex silicates, such as calcium aluminates and hydrated calcium silicates.^25^ These compounds have stronger and more stable chemical bonds, which limits the release of ions in solution.^26^ Their fine particles result in a greater surface area and a smaller quantity of ions available for release.^27^ Furthermore, calcium silicate-based materials form a layer of calcium hydroxide when in contact with biological fluids, which also helps with the controlled release of ions.^28^ Calcium hydroxide-based materials have a layer of hydroxide ions and calcium ions bound together by electrostatic forces. This relatively less rigid crystalline structure and less intense electrostatic forces make it easier for ions to dissociate and release intro the aqueous medium.^29^

Moreover, as mentioned above, BCT has a resinous vehicle in its composition, which prevents the medication from setting,^4^ resulting in lower release of OH- ions, less alkalinization and, consequently, less antimicrobial potential. Calcium hydroxide CAL paste has polyethylene glycol 400 as a vehicle, providing greater release of OH- ions, which occurs slowly and continuously, increasing the pH, time of action, solubility and antimicrobial effect.^30^In addition, viscous vehicles have lower surface tension, allowing greater diffusion of the medication through the bacterial cell membrane^24^ and through the dentinal tubules.^31^

BCT shows lower antibacterial activity when compared with calcium hydroxide pastes CAL and UltraCal XS due to the lower availability of calcium hydroxide resulting from the hydration reaction.^9^ The association of 50% or more of CAL with BCT medication was able to promote greater solubility, alkalinization potential and more effective intratubular disinfection against *E. faecalis*. When Calcium silicate-based materials are in the presence of moisture, they form Ca(OH)_2_, and dissociation into OH^-^ and Ca^++^ ions also occurs.^4^Therefore, a higher level of availability of ions can provide more intense antibacterial activity.^9^

The solubility assessment in the present study was based on the American National Standards Institute/American Dental Association (ANSI/ADA) specifications for endodontic sealers, since there are no specific standards for intracanal medications. The solubility of calcium hydroxide-based pastes was evaluated after immersion in distilled water.^13,24^ Another suggestion would be to perform immersion in phosphate buffered saline (PBS) to simulate clinical application, which could interfere with the solubility and volumetric change of bioceramic materials.^32^

The association of Ca(OH)_2_ paste with different vehicles and additives can influence its solubility.^13,24^ In the present study, CAL showed greater solubility than BCT, and its association with CAL promoted an increase in the solubility of bioceramic medication. These results suggest that solubility, increase in pH, and antimicrobial activity must be related. The viscous vehicle present in the CAL paste may have provided higher solubility values and contributed to the release of OH- ions, increasing the pH and intratubular disinfection of the BCT bioceramic. However, future studies are needed to assess whether the addition of calcium hydroxide to BCT would maintain the bioactive properties of this medication.

*E. faecalis* is frequently observed in cases of endodontic treatment failure^33^ and used to represent resistant microbiota^34^, being able to survive in conditions of nutritional restriction, alkaline environment, penetrate into the dentinal tubules and form biofilm.^20,35,36^Intratubular decontamination by intracanal medication was evaluated by CLSM, using *E. faecalis* for the contamination of bovine dentin tubules, due to its similarity to human dentin.^37^This methodology allows the identification of living and dead cells within the dentinal tubules.^33,37^The association of calcium hydroxide paste with the bioceramic medication BCT , at a concentration of 50% or more, demonstrated higher levels of intratubular disinfection against *E. faecalis*. Calcium hydroxide paste demonstrated the ability to significantly reduce *E. faecalis* CFU^-1^ values after dentin contamination.^17,24,37^The more extensive release of OH- ions provided high reactivity in the bacterial cytoplasmic membrane, decreasing cellular biological activity.^38^ It also provided increased alkalinity, affecting bacterial proliferation and survival, inhibiting enzyme activity, and affecting metabolism and microbial growth.^39^

Furthermore, another factor that may have influenced the better intratubular decontamination by the association of medications with 50-75% CAL was dentin penetrability. BCT contains a resinous vehicle and Ultracal® XS contains an aqueous vehicle. Bio C Temp showed absence of dentinal tubule penetration.^4^Whereas the presence of a viscous vehicle in the intracanal medication improved its intratubular penetration.^31^Therefore, the association of Ca(OH)_2_ medication containing a viscous vehicle may have favored the intratubular penetration of the bioceramic medication. Future evaluations are necessary to verify the interference of the combination of medications in penetrability.

The major cause of endodontic treatment failure I known to be persistent infection. Moreover, the use of intracanal medication is essential to complement the preparation of root canals, provided that has a broad antimicrobial spectrum and induces the repair of periapical lesions.^25^ At present several intracanal medications are available for endodontic treatment. However, no medication has all the ideal characteristics.^40^ Hence the importance of the present study in proposing the association of a calcium hydroxide-based paste routinely used in clinical practice, with a new bioceramic medication to improve its properties by associating the bioactivity of the bioceramic with the antimicrobial effect of calcium hydroxide. Although the results demonstrated greater alkalinization and antimicrobial activity against endodontic infections, additional studies are needed to evaluate the impact of the associations on the bioactivity of Bio-C Temp. Furthermore, considering the results obtained in the disinfection of the root canal system, calcium hydroxide paste continues to be an excellent option.

## Conclusion

Combinations of Bio-C® Temp with 50% or more of calcium hydroxide paste medication provided greater alkalinization, increased solubility, and increased intratubular disinfection of the bioceramic medication.

## Data Availability

The datasets generated during and/or analyzed during the current study are available from the corresponding author on reasonable request.
